# Multiscale Investigation of Interfacial Behaviors in Rubber Asphalt–Aggregate Systems Under Salt Erosion: Insights from Laboratory Tests and Molecular Dynamics Simulations

**DOI:** 10.3390/ma18204746

**Published:** 2025-10-16

**Authors:** Yun Li, Youxiang Si, Shuaiyu Wang, Peilong Li, Ke Zhang, Yuefeng Zhu

**Affiliations:** 1 School of Mechanical and Electrical Engineering, Anhui Jianzhu University, Hefei 230601, China; liyunfxc@163.com; 2 School of Civil Engineering and Architecture, Anhui University of Science & Technology, Huainan 232001, China; 15055415306@163.com (Y.S.); 13135545204@126.com (S.W.); 3 School of Highway, Chang’an University, Xi’an 710064, China; lipeilong@chd.edu.cn; 4 School of Business, Fuyang Normal University, Fuyang 236041, China; 5 Anhui Transport Consulting & Design Institute Co., Ltd., Hefei 230088, China; 6 College of Engineering, Computer Science, and Construction Management, California State University-Chico, Chico, CA 95929, USA; zhuyuefengfeng@126.com

**Keywords:** rubber asphalt-aggregate, salt erosion, interfacial behaviors, laboratory test, molecular dynamics simulations

## Abstract

Deicing salt effectively melts ice and snow to maintain traffic flow in seasonal freezing zones, but its erosion effect compromises the water stability and structural integrity of asphalt pavements. To comprehensively explore the impacts of salt erosion on the interfacial behaviors of rubber asphalt–aggregate systems, this study developed a multiscale characterization method integrating a macroscopic mechanical test, microscopic tests, and molecular dynamics (MD) simulations. Firstly, laboratory-controlled salt–freeze–thaw cycles were employed to simulate field conditions, followed by quantitative evaluation of interfacial bonding properties through pull-out tests. Subsequently, the atomic force microscopy (AFM) and Fourier transform infrared spectrometer (FTIR) tests were conducted to characterize the microscopic morphology evolution and chemical functional group transformations, respectively. Moreover, by combining the diffusion coefficients of water molecules, salt solution ions, and asphalt components, the mechanism of interfacial salt erosion was elucidated. The results demonstrate that increasing NaCl concentration and freeze–thaw cycles progressively reduces interfacial pull-out strength and fracture energy, with NaCl-induced damage becoming limited after twelve salt–freeze–thaw cycles. In detail, with exposure to 15 freeze–thaw cycles in 6% NaCl solution, the pull-out strength and fracture energy of the rubber asphalt–limestone aggregate decrease by 50.47% and 51.57%, respectively. At this stage, rubber asphalt exhibits 65.42% and 52.34% increases in carbonyl and sulfoxide indexes, respectively, contrasted by 49.24% and 42.5% decreases in aromatic and aliphatic indexes. Long-term exposure to salt–freeze–thaw conditions promotes phase homogenization, ultimately reducing surface roughness and causing rubber asphalt to resemble matrix asphalt morphologically. At the rubber asphalt–NaCl solution–aggregate interface, the diffusion of Na^+^ is faster than that of Cl^−^. Meanwhile, compared with other asphalt components, saturates exhibit notably enhanced mobility under salt erosion conditions. The synergistic effects of accelerated aging, salt crystallization pressure, and enhanced ionic diffusion jointly induce the deterioration of interfacial bonding, which accounts for the decrease in macroscopic pull-out strength. This multiscale investigation advances understanding of salt-induced deterioration while providing practical insights for developing durable asphalt mixtures in cold regions.

## 1. Introduction

Asphalt mixtures are an important paving material in road engineering, and their application scale in the surface layers of high-grade highways has been expanding in recent years. With severe snow conditions in winter in northern China, deicing salt and snowmelt are widely used to guarantee the safety and quality of road traffic. However, the erosion of snowmelt water and salt seriously damages the durability of asphalt pavements [1,2,3]. A substantial amount of research has been conducted on how salt-containing materials like deicing salts and snowmelt agents affect the mechanical properties of asphalt mixtures, and studies have verified that salt crystallization erosion intensifies water-induced water damage [4,5]. According to Xiong et al. [6], the penetration of salt solutions into voids and cracks within asphalt mixtures leads to crystallization erosion, which significantly contributes to performance degradation. Zhang et al. [7] conducted fraction tests and atomic force microscopy (AFM) to simulate the harmful effects of a salt-erosive environment on asphalt binders, and found that the surface roughness and honeycomb structure are reduced to varying degrees. In addition, erosion from salt diminishes the splitting strength of asphalt mixtures and severely disrupts the moisture stability [8]. As the concentration of chloride solution goes up, the resistance of asphalt mixtures to rutting and cracking also declines [9,10]. For example, Hu et al. [11] found that chloride-based snowmelt agents impair the rheological characteristics and creep behavior of asphalt binders via penetration and ductility tests. Using the multiple stress creep recovery (MSCR) test, Busang et al. [12] investigated the mechanical properties of asphalt binders in the presence of salt. The results show that when the salt concentrations are 1%, 3%, and 5%, the shear strains of the immersed samples are 378, 395, and 421, respectively. Correspondingly, the recovery rates are 10.1%, 9.1%, and 6.6%, and the irrecoverable creep flexibilities are 208 kPa^−1^, 223 kPa^−1^, and 257 kPa^−1^. Furthermore, Gilani and Behbahani et al. [13,14] explored how different deicers and sodium chloride affect the water sensitivity and fatigue damage of asphalt mixtures. Mackiewicz et al. [15] immersed asphalt mixture samples in water and salt solutions, analyzed the variations in stiffness modulus, and established a relationship between mass variation and the stiffness modulus.

Undoubtedly, the deterioration of asphalt pavements due to deicing salts has emerged as a pivotal research area in cold region engineering, especially given the widespread use of chloride-based deicers. While numerous studies have documented macroscopic signs of salt-induced damage, such as accelerated peeling, increased cracking, and reduced skid resistance, the underlying physicochemical mechanisms driving these deterioration processes remain poorly understood at the microscopic level [16,17,18]. Currently, research primarily relies on traditional mechanical testing methods. Although valuable for performance assessment, these methods fail to capture the complex interfacial phenomena occurring between asphalt binders and mineral aggregates in salt–freeze–thaw environments. This knowledge gap is particularly critical because the durability of asphalt mixtures is fundamentally determined by interfacial molecular-scale interactions between asphalt and aggregates, where salt crystallization, chemical reactions, and moisture transport jointly contribute to the progressive accumulation of damage. Traditional macroscopic approaches are inadequate in three key aspects: first, the microscopic morphological evolution of asphalt surfaces during salt exposure cannot be effectively characterized; second, these methods fail to capture the chemical transformation of functional groups in asphalt binders; third, they are unable to reveal the dynamic interactions among water, salt, and the binder at the interface, all of which are decisive factors for the long-term properties of asphalt mixtures.

Fortunately, the AFM test enables effective characterization of the microscopic morphology of the asphalt surface, while the Fourier transform infrared spectrometer (FTIR) can acquire the variations in asphalt functional groups. Additionally, a molecular dynamics simulation (MD) can elucidate the microstructural changes in asphalt binders at the molecular level [19,20,21,22,23,24]. Yi et al. [25,26] used atomic force microscopy to examine the microscopic morphology and adhesion properties of various asphalts, concluding that the adhesion between asphalt and aggregates is primarily tied to physical adhesion. Menapace et al. [27] examined the variations in asphalt microstructure after accelerated aging tests via AFM, revealing notable differences depending on moisture presence. Nivedya et al. [28] discussed the moisture’s impact on asphalt microstructure and found significant differences between asphalt mixed with a reactivator and the control group. Ma et al. [29] utilized AFM to study the effects of aging and regeneration on asphalt microstructure and adhesion performance, with macroscale tests validating the enhanced adhesive force between recycled asphalt and aggregates. Hu et al. [30] utilized AFM and infrared spectroscopy examine the influence of graphene oxide on SBS-modified asphalt and elucidated its interfacial enhancement mechanism. Zhang et al. [31] employed an infrared spectrometer to analyze how temperature, humidity, and salt environment affect the chemical constituents of asphalt mortar, showing that the salt accelerates its aging in an aqueous solution. Zou et al. [32] explored the effects of aqueous solutions on asphalt’s physical and chemical properties, discovering that as the immersion time in the aqueous solution prolongs, the indexes of functional groups, specifically the carbonyl and sulfoxide indexes, increase.

Meanwhile, Zhang et al. [33] assessed the erosive impacts of various solutions on the interface of asphalt and aggregates via MD simulation, and found that acidic and alkaline solutions reduce asphalt adhesion. Sonibare et al. [34] used vegetable oil as an asphalt modifier and conducted MD simulations on the fatty acid component and asphalt models. Wu et al. [35] elucidated the diffusion properties of emulsified asphalt on aggregate and elucidated the roles played by water, emulsifiers, and asphalt in the process of adsorption from a molecular standpoint. Cao et al. [36,37,38] observed the microscopic characteristics of aged and regenerated asphalt using MD and investigated their interaction mechanism. Furthermore, Luo et al. [39,40] employed MD technology to reveal the adhesive mechanism of asphalt and aggregate under oxidative aging. With the help of the MD technique, Jiao et al. [41] explored the degradation behavior of rubber particles, as well as the interfacial adhesion between the rubber asphalt and aggregates under the impact of moisture. The results illustrate that the incorporation of rubber powder in asphalt significantly improves the resistance of asphalt mixtures to moisture damage. The aforementioned research further verified the feasibility of employing AFM, FTIR, and MD simulation approaches to explore the microscopic characteristics and molecular behavior of asphalt. This offers a crucial reference for investigating the interface damage behaviors between rubber asphalt and aggregates subjected to a salt–freeze–thaw environment.

To fundamentally understand the interfacial deterioration mechanisms in rubber asphalt–aggregate systems under salt erosion, this study undertakes a multiscale exploration of these mechanisms in such systems exposed to a combined salt–freeze–thaw environment. This particular area has received relatively limited attention in the existing literature. In contrast to previous research, which predominantly concentrated on macroscopic properties or the impacts of single factors, this research takes a distinctive approach. It seamlessly integrates macro-mechanical tests, micro-characterization techniques, and MD simulations. This comprehensive integration enables a correlation between the degradation of bonding properties and the evolution of microscopic morphology, chemical composition, and molecular-level interfacial interactions. This work not only provides novel insights into the physicochemical mechanisms of salt-induced damage but also establishes a multiscale framework for evaluating and improving the durability of asphalt mixtures in cold regions.

## 2. Materials and Methods

### 2.1. Raw Materials

**Figure 1:**
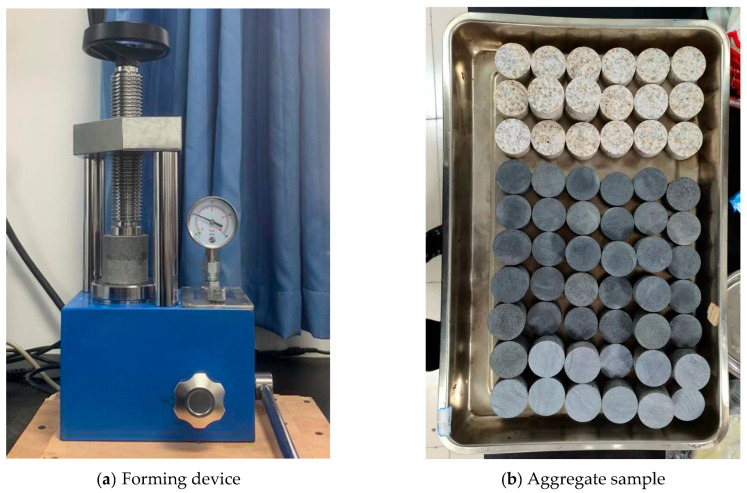
Preparation of pull-out samples [46].

In the laboratory, rubber asphalt was prepared via a controlled blending process. The 60-mesh recycled crumb rubber was incorporated into SK70# matrix asphalt at a 20% mass. The performance parameters of both the matrix and rubber asphalt were tested in the laboratory. The test results, as presented in Table 1, comply with the requirements of relevant standards [42,43,44,45]. The asphalt–aggregate specimens were made from limestone, basalt, and granite stones. The main chemical constituents of these aggregates were also tested, as presented in Table 2. The asphalt–aggregate specimens were prepared with the following procedures [46]: (1) Core preparation: Cylindrical samples (Ø50 × 25 mm) were cored from quarry rocks. Then the samples were boiled in water for 15 min to clean the surface, and dried at 110 °C for 24 h. (2) Interface construction: Samples were pre-heated at 135 °C for 4 h. Then 1.5 g of hot rubber asphalt was precisely applied, and the upper and lower samples were coaxially aligned. (3) Specimen formation: The samples were compressed in a forming device under 96.5 kPa for 5 min to form uniform interfacial films, as shown in Figure 1, followed by 24 h ambient curing to ensure stability.

### 2.2. Test Method

#### 2.2.1. Simulation of the Salt–Freeze–Thaw Cycles

A laboratory investigation of rubber asphalt and asphalt–aggregate subjected to salt–freeze–thaw cycles was carried out to evaluate the deterioration mechanism. Four concentration gradients of NaCl solutions (0%, 3%, 6%, and 9%) were configured to systematically simulate the deicing salt exposure scenarios. Prior to the treatment, the specimens were preconditioned through 12 h immersion in the designated salt solution to achieve complete saturation. The experimental protocol included exposure to 3, 6, 9, 12, and 15 freeze–thaw cycles (−20 °C to 30 °C) in salt solution, with control specimens maintained without freeze–thaw cycles [47]. Each complete cycle comprised (1) 11–12 h of freezing at −20 °C in a climate-controlled chamber, followed by (2) 12–13 h of thawing in temperature-regulated NaCl solution at 30 °C.

#### 2.2.2. Macroscopic Pull-Out Test of Rubber Asphalt–Aggregate

**Figure 2:**
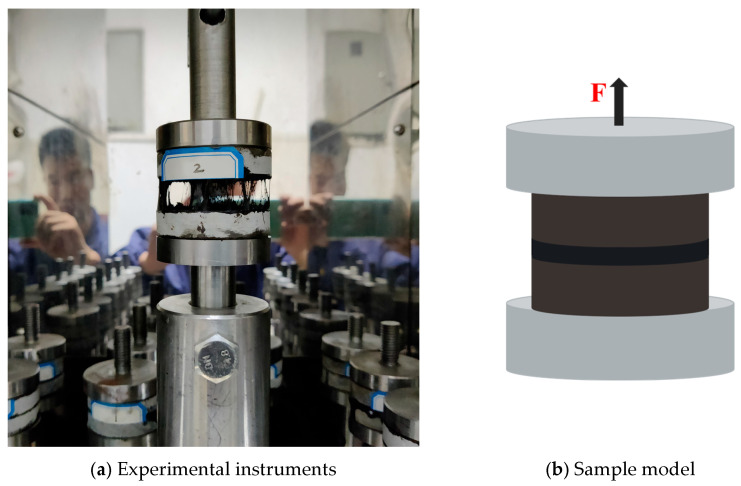
Interfacial pull-out test between rubber asphalt and aggregate.

After subjecting specimens to various freeze–thaw cycles in 0%, 3%, 6%, and 9% NaCl solutions, the interfacial pull-out tests between asphalt and aggregate were conducted, as illustrated in Figure 2. The interfacial pull-out strength between rubber asphalt and aggregate was determined at 25 °C with a loading rate of 5 mm/min. The number of parallel samples for the pull-out test is 3. The indexes of pull-out strength and fracture energy were adopted as indexes to evaluate the interfacial bonding performance between asphalt and aggregate, and their calculation formulas are provided in Equations (1)–(3).

(1) $$P=\frac{F}{A}$$

where *P* represents the pull-out strength, MPa; *F* represents the destructive force in the course of the experiment, N; and *A* is the cross-sectional area of the pull-out specimen, 19.625 cm^2^.

(2) E=∫P(x)dx

where *E* is the fracture energy, MJ/m^2^; *P*(*x*) denotes the load–displacement curve of the pull-out test between rubber asphalt and aggregate.

According to Equations (1) and (2), the pull-out strength and fracture energy of different asphalt and aggregates under salt erosion conditions can be calculated. To characterize the variations in pull-out strength and fracture energy more accurately and thus describe the salt erosion resistance, the loss rate of pull-out strength or fracture energy is defined as given in Equation (4).

(3) $$\theta =\frac{P-P_{i}}{P}\times 100\%$$

where *θ* is the loss rate, %; *P* represents the pull-out strength or fracture energy of the specimen without freeze–thaw cycles, MPa; *P_i_* is the evaluation index of the specimen after freeze–thaw cycles.

#### 2.2.3. Characterization of Microscopic Properties for Rubber Asphalt

##### AFM Test

AFM was adopted to characterize the surface topographic evolution of rubber asphalt specimens exposed to salt–freeze–thaw cycles. All measurements were implemented in tapping mode, with six representative areas (20 μm × 20 μm) randomly scanned per specimen. The number of parallel samples for the AFM test is 3. Quantitative surface roughness analysis was conducted using Nanoscope Analysis software (v1.5), wherein the average roughness (*Ra*) and root mean square roughness (*Rq*) were adopted as key morphological evaluation parameters, as shown in Equations (4) and (5).

(4) $$R_{a}=\frac{1}{N}\sum_{j=1}^{N}\left|Z_{j}\right|$$

(5) $$R_{q}=\sqrt{\frac{\iint {\left[h(x,y)-h_{0}\right]}^{2}d_{S}}{\iint d_{S}}}$$

Here, *Ra* refers to average roughness, nm; *Rq* denotes root mean square roughness, nm; *Z_j_* represents the elevation of test points, nm; *N* denotes the total quantity of test points; *h (x, y)* refers to the height function, nm; *h*_0_ stands for the reference height, nm; *S* represents the scanning area of 20 μm × 20 μm.

##### FTIR Test

FTIR was employed to characterize the evolution of functional groups in rubber asphalt after combined salt–freeze–thaw cycles. Measurements were taken with an SYD-0673M spectrometer under these parameters: a spectral resolution of 4 cm^−1^, 32 accumulated scans, and a wavenumber range from 4000 to 600 cm^−1^. The samples were prepared by the KBr tableting method. Anhydrous KBr powder was finely ground in an agate mortar and pressed into wafers using a tableting machine. The equipment of SYD-0673M spectrometer and tableting machine was produced by Shanghai Changji Geological Instrument Co., LTD in China. Before measuring samples, background spectra were obtained by scanning a blank KBr wafer on the magnetic sample holder. For analysis, molten asphalt was evenly coated on freshly prepared KBr wafers to form homogeneous asphalt films. All wafers were stored in a desiccator with anhydrous calcium sulfate to avoid moisture absorption before FTIR measurements. The number of parallel samples for the FTIR test is 3. Following the test completion, absorbance spectroscopy was used to compare the chemical composition changes in rubber asphalt after different processing methods. OMNIC 8.2 software was used for spectral data processing, focusing on characteristic absorption bands of carbonyl, sulfoxide, aromatic rings, and aliphatic chains. Functional group indexes were quantitatively evaluated using Equations (6)–(9) [48], facilitating a systematic assessment of chemical changes in rubber asphalt induced by salt–freeze–thaw cycles.

(6) $$I_{C=O}=A_{C=O}/\sum A$$

(7) $$I_{S=O}=A_{S=O}/\sum A$$

(8) $$I_{Aromaticity}=A_{1600{\mathrm{cm}}^{-1}}/\sum A$$

(9) $$I_{Aliphatic}=(A_{1467{\mathrm{cm}}^{-1}}+A_{1379{\;\mathrm{cm}}^{-1}})/\sum A$$

In the formula, *I_C=O_* stands for the carbonyl index, *I_S=O_* refers to the sulfoxide index, *I_Aromaticity_* represents the aromatic index, and *I_Aliphatic_* is the aliphatic index. ΣA denotes the total area of the C-H peaks in the infrared spectral absorption range from 2000 to 600 cm^−1^. *A_C=O_* represents the area of the carbonyl peak at around 1700 cm^−1^, and *A_S=O_* signifies the area of the sulfoxide peak at approximately 1017 cm^−1^. *A*_1600cm_^−1^ represents the peak area of the aromatic ring near 1600 cm^−1^, *A*_1467cm_^−1^ denotes the peak area of the aliphatic chain around 1467 cm^−1^, and *A*_1379cm_^−1^ stands for the peak area of the branched-chain alkane near 1379 cm^−1^.

#### 2.2.4. Simulation of Molecular Behavior at the Asphalt–Aggregate Interface

##### Interfacial Modeling of Asphalt and Aggregate

The isothermal isobaric ensemble (NPT) and canonical ensemble (NVT) were adopted according to the characteristics and needs of the simulation [49]. Commonly, there are three force field options for modeling asphalt molecules: GAFF, COMPASS, and ReaxFF. This work focuses on the interfacial performance of rubber asphalt and aggregates, including organic and inorganic molecules [50], and the COMPASS force field was consequently chosen.

##### Rubber Asphalt Molecular Model

**Figure 3:**
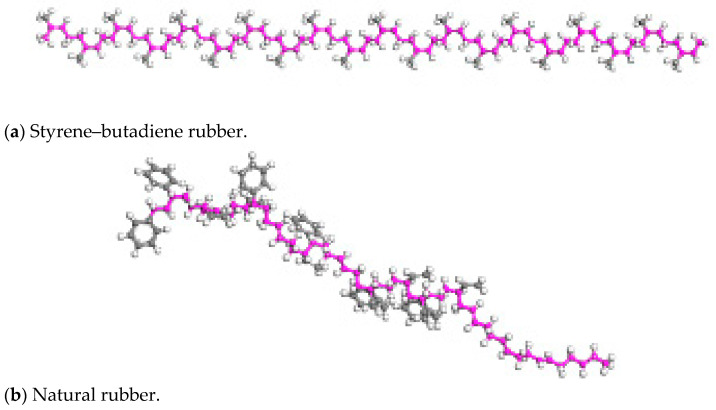
Rubber molecular model.

Since the main components of rubber powder in rubber asphalt are styrene–butadiene rubber and natural rubber, a model consisting of 80% styrene–butadiene rubber and 20% natural rubber was chosen to represent the rubber phase, with the rubber molecular model illustrated in Figure 3.

**Figure 4:**
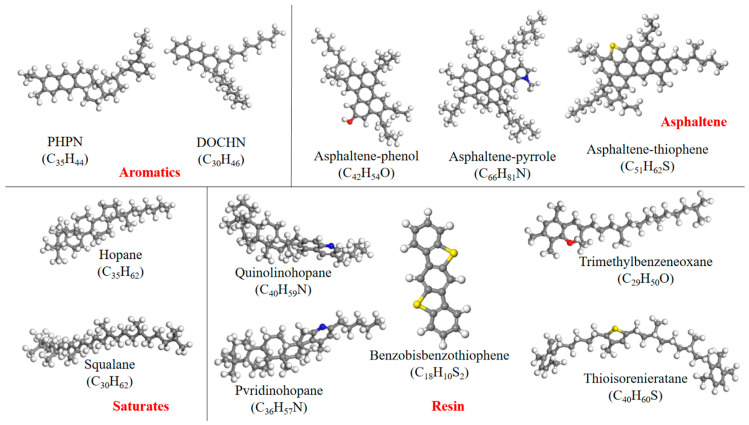
Twelve-molecule model of asphalt.

Matrix asphalt has a complex material composition, encompassing various hydrocarbons, non-hydrocarbons, and impurities. This complexity makes it challenging to simulate its properties using a single molecular structure. To conduct a detailed and dynamic simulation of asphalt, the molecular assembly structure method was employed to build the molecular model [51,52]. In this study, in combination with rubber molecules, an asphalt model with 4 components and 12 molecules was selected to develop the models of matrix asphalt and rubber asphalt. This 4-component, 12-molecule asphalt model is presented in Figure 4.

**Figure 5:**
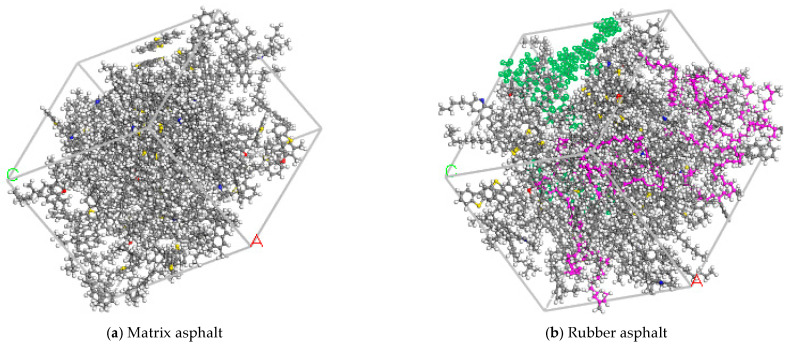
Molecular dynamics model of matrix asphalt and rubber asphalt.

The modeling process for rubber asphalt was as follows: Firstly, the amorphous unit module was selected to create the molecular models for matrix asphalt and rubber asphalt in MS software 2020. Secondly, the Forcite module was employed to refine the conformations of these models. Ten annealing treatments were carried out within the temperature range of 300 K to 500 K to eliminate unstable structures in the asphalt models, ensuring the stability of the molecular conformations. Finally, a 100 ps dynamics simulation was run to further confirm the stability and reliability of the asphalt models. The asphalt model is presented in Figure 5.

##### Aggregate Molecular Model

The aim of this work is to simulate and assess the interfacial behaviors between rubber asphalt and aggregates. Two widely used engineering materials, basalt and limestone, were chosen as aggregate models. The main chemical constituents of these mineral aggregates are SiO_2_, CaO, Fe_2_O_3_, and Al_2_O_3_. In this study, SiO_2_ and CaO were selected to stand for acidic and alkaline aggregates, respectively. The unit models of SiO_2_ and CaO were cut along the (1, 0, 0) plane and introduced into supercells to obtain the aggregate interface.

##### Interfacial Model Between Asphalt and Aggregate

**Figure 6:**
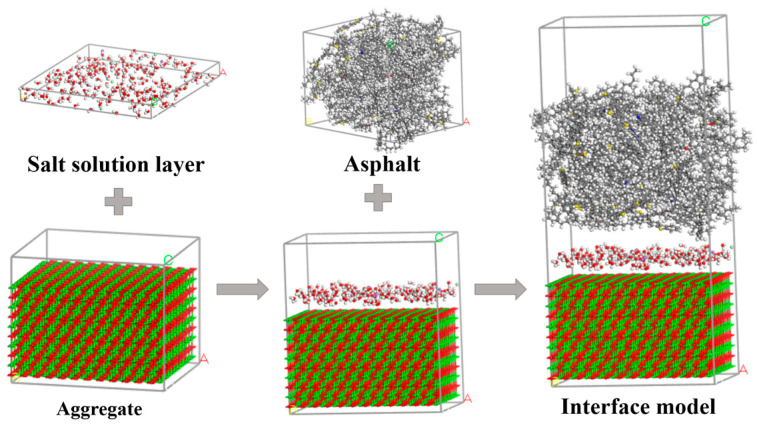
Molecular dynamics modeling of the asphalt–salt solution–aggregate.

Under the coupled effect of salt, freeze, and thaw, interfacial damage occurs between asphalt and aggregate. Salt and water compete with asphalt for adsorption points, which affects the interfacial bonding. Some salt solution molecules adsorbed on the surface of aggregate replace the original asphalt molecules. Accordingly, a salt solution layer was added between the asphalt and aggregate in line with their model to mimic such interfacial damage. The degree of interfacial damage was controlled by adjusting the quantity of water molecules in the salt solution layer. In subsequent simulations, the number of water molecules was set in gradients of 100, 150, 200, 250, and 300. Correspondingly, salt solution layers with concentrations of 0% and 6% were also set, as shown in Figure 6.

##### Evaluation Indicators for Interfacial Behavior

Mean squared displacement (MSD) in molecular dynamics simulations is a crucial physical index that describes the time-dependent diffusion behavior of molecules. It serves as a key parameter for studying material properties (such as diffusion coefficients) and dynamic behavior. MSD reflects the average change in particle position over time and can quantify the movement of a molecule within a specific period, as shown in Equation (10). The formula for determining the diffusion coefficient is presented in Equation (11) [53,54].

(10) $$MSD=\langle {\left|r(t)-r(0)\right|}^{2}\rangle$$

where *r*(*t*) denotes the particle position corresponding to time *t*, *r* (0) represents the initial particle position, *r*(*t*)*–r*(0) stands for the particle’s shift from the initial moment to time *t*, and *<·>* stands for the averaging algorithm applied to all particles and a sufficient number of independent time origins.

(11) $$D=\frac{1}{6}\;\underset{t\to \infty }{\lim}\frac{dMSD}{dt}$$

In the above equation, the slope is obtained by calculating the ratio of MSD to time, with units of 10^−8^ cm^2^/s, and the diffusion coefficient (*D*) is expressed as 1/6 of the curve slope.

To analyze the bonding at the asphalt–aggregate interface in salt-eroded environments, interfacial energy is used as an evaluation index. A higher interfacial energy indicates better bonding properties between the two materials. The interfacial energy of the rubber asphalt–aggregate system can be computed through Equation (12) [55].

(12) $$W_{\mathrm{int}er}=(E_{total}-(E_{ra-w}+E_{agg-w})+E_{w})/A$$

Here, *W_inter_* denotes the interfacial energy, MJ/m^2^; *E_total_* is the total energy of the asphalt–solution–aggregate system, mJ; *E_ra-w_* stands for the energy of the asphalt–solution model, mJ; *E_agg-w_* represents the energy of the aggregate–solution model, mJ; *E_w_* is the energy of the solution system, mJ; *A* signifies the area of the aggregate, m^2^.

## 3. Results and Discussion

### 3.1. Interfacial Pull-Out Properties Between Rubber Asphalt and Aggregate

#### 3.1.1. Pull-Out Properties Under Different Aggregate Types

**Figure 7:**
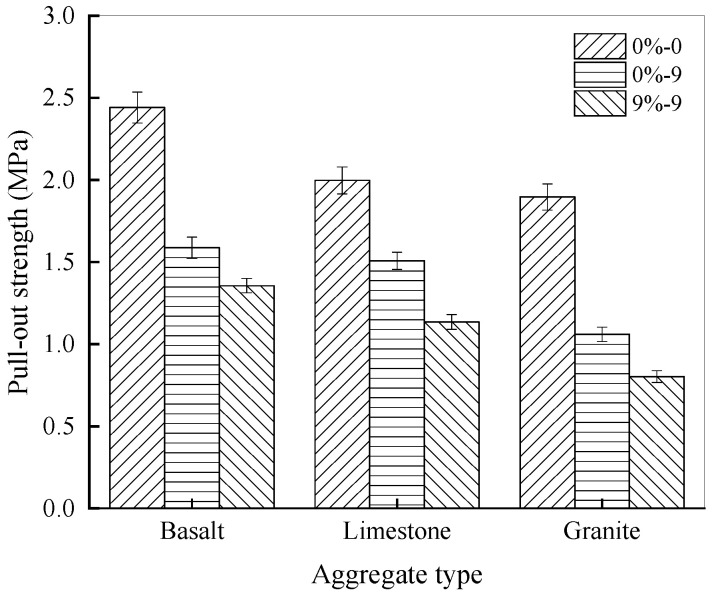
Pull-out strength under different aggregate types.

To explore the impact of aggregate type on interfacial bonding between rubber asphalt and aggregate, basalt, limestone, and granite were selected to conduct the pull-out tests after nine freeze–thaw cycles in which samples were exposed to NaCl solutions with concentrations of 0% and 9%. Additionally, a pull-out test was carried out for the control group that received no salt erosion treatment. Figure 7 presents the pull-out strength across different aggregate types.

As illustrated in Figure 7, prior to exposure to salt–freeze–thaw cycles, the interfacial pull-out strengths of basalt, limestone, and granite with rubber asphalt are 2.441 MPa, 1.997 MPa, and 1.896 MPa, in that order. However, after nine freeze–thaw cycles in which samples are exposed to 0% NaCl solution, the pull-out strengths become 1.587 MPa, 1.507 MPa, and 1.060 MPa, representing reductions of 34.99%, 24.51%, and 44.10%, respectively. Notably, after the freeze–thaw treatment in pure water, granite experiences the most significant reduction in pull-out strength, while limestone has the smallest reduction. Throughout the freeze–thaw process, the order of pull-out strength remains basalt > limestone > granite. This disparity is primarily attributable to the quartz content in the aggregates. The aggregates with higher quartz content, like granite, exhibit poor bonding with asphalt. Moreover, following nine freeze–thaw cycles conducted in a 9% NaCl solution, the pull-out strengths of basalt, limestone, and granite with rubber asphalt are 1.356 MPa, 1.135 MPa, and 0.802 MPa, respectively. It is noteworthy that in a salt-eroded environment, the pull-out strength between limestone aggregates and rubber asphalt is higher than that between granite aggregates. Comparing the decline in pull-out strength, the strength between limestone and asphalt decreases by 43.15%, while that of granite decreases by 57.72%, further confirming that alkaline aggregates bond more effectively with rubber asphalt than acidic aggregates, which is consistent with the conclusion obtained by Huang et al. [56].

**Figure 8:**
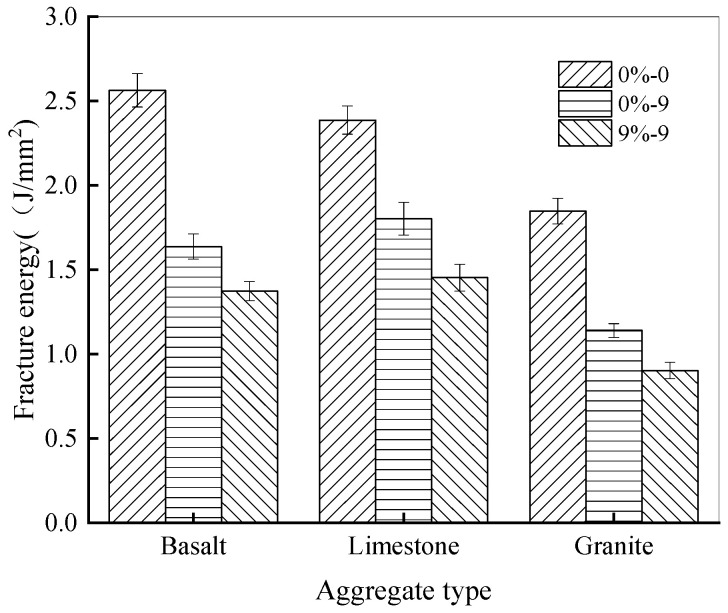
Fracture energy under different aggregate types.

The interfacial fracture energy between rubber asphalt and basalt, limestone, and granite appears in Figure 8.

As shown in Figure 8, for basalt, granite, and limestone aggregates, the fracture energy of rubber asphalt and aggregates varies significantly without salt–freeze–thaw coupling, with the values of 2.563 MJ/m^2^, 2.386 MJ/m^2^, and 1.847 MJ/m^2^, respectively. Following a set number of freeze–thaw cycles in salt solution, the fracture energy demonstrates a remarkable decline. For instance, after nine freeze–thaw cycles while exposed to 9% NaCl solution, the fracture energy of basalt, limestone, and granite with rubber asphalt decreases by 46.43%, 39.09%, and 51.12%, respectively. Among them, the bonding of rubber asphalt to basalt and limestone is much stronger than to granite. This is mainly because granite, an acidic aggregate with high SiO_2_ content, is prone to interface segregation with rubber asphalt after the salt–freeze–thaw process [57]. The combined action of water, salt, and freeze–thaw generates significant crystallization pressure and osmotic pressure within the asphalt–aggregate interface. This process disrupts the physical and chemical bonds, leading to accelerated moisture damage, a mechanism that has been documented in previous studies on asphalt mixture deterioration [58]. Compared with the control group, when the number of cycles grows, the interfacial spalling between rubber asphalt and granite becomes more severe. Consequently, the fracture energy between rubber asphalt and granite decreases more rapidly when subjected to salt–freeze–thaw cycles. On the contrary, basalt and limestone exhibit superior physical and chemical adhesion to rubber asphalt. After the combined effect of salt–freeze–thaw, their fracture energy decreases at a slower rate than that of granite, resulting in greater resistance to water damage in a salt-eroded environment [59]. In the subsequent analysis, limestone aggregates were selected to investigate how the salt solution concentration and freeze–thaw cycles influence outcomes.

#### 3.1.2. Pull-Out Properties at Different Salt Solution Concentrations

**Figure 9:**
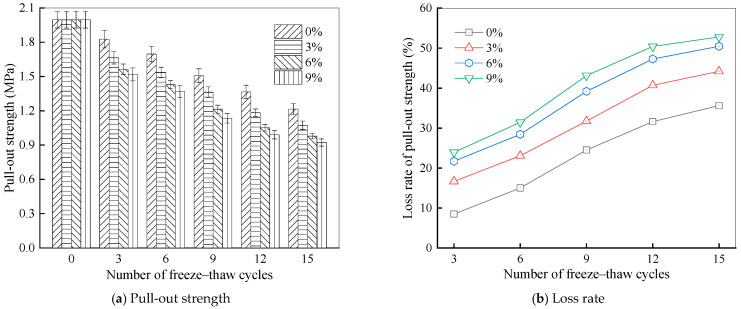
Pull-out strength under different salt solution concentrations.

To verify the impact of salt solution concentrations on the interfacial bonding behavior of rubber asphalt and limestone aggregates, pull-out tests were performed on specimens treated with 0%, 3%, 6%, and 9% salt solution concentrations. The test results are shown in Figure 9.

As depicted in Figure 9, the pull-out strengths of specimens subjected to the salt–freeze–thaw coupling effect all decrease to varying extents. Specifically, after being treated in a 3% salt solution, the pull-out strength falls by 16.67%, 23.09%, 31.75%, 40.71%, and 44.21%, corresponding to 3, 6, 9, 12, and 15 cycles, respectively. Moreover, with the same salt solution, the pull-out strength of the samples drops sharply as the number of action times rises. When the number of cycles exceeds 12, this decline rate moderates. This trend is consistent with the findings of [60], who reported a similar asymptotic decay pattern in bond strength after a critical number of freeze–thaw cycles, suggesting a damage saturation effect. In the salt–freeze–thaw environment, the sodium ions cause rubber asphalt to undergo “salt aging”. This phenomenon impairs the adhesive properties of rubber asphalt, thus lowering the pull-out strength. Nevertheless, once the salt–freeze–thaw cycles reach a certain number, the impact of NaCl-induced freeze–thaw crystallization on both the extent of asphalt film damage at the specimen interface and the aging process becomes restricted. As a result, the decline in pull-out strength of the rubber asphalt–aggregate system decelerates and eventually approaches a stable state.

Furthermore, under identical freeze–thaw cycle counts, the decline in interfacial adhesion performance between rubber asphalt and aggregate intensifies as NaCl solution concentration rises. This indicates that the NaCl solution accelerates moisture-induced damage at the interface of asphalt and aggregates during freeze–thaw processes. However, when the NaCl concentration exceeds 6%, the reduction rate of pull-out strength in the rubber asphalt–aggregate system gradually diminishes with further increases in concentration. For example, after 15 action times, the pull-out strength of rubber asphalt–aggregate samples treated in 0%, 3%, 6%, and 9% NaCl solutions decreases by 35.63%, 44.21%, 50.47%, and 52.81%, respectively. This observation stems from the increasing surface tension of the NaCl solution at higher concentrations. Once the NaCl concentration surpasses a critical threshold, its ability to penetrate the rubber asphalt–aggregate interface becomes limited, thereby slowing the decrease rate of pull-out strength.

**Figure 10:**
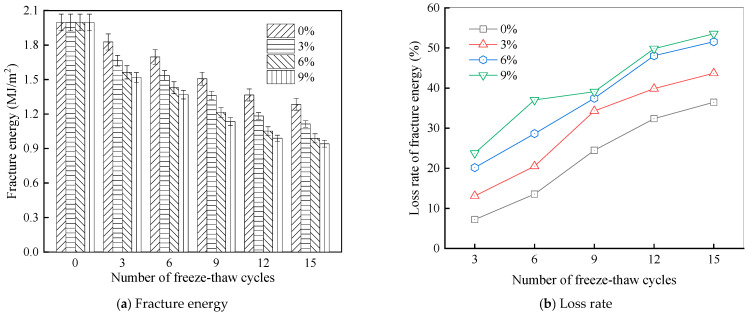
Fracture energy under different salt solution concentrations.

Figure 10 presents the fracture energy between rubber asphalt and aggregates under different salt–freeze–thaw processes.

As shown in Figure 10, as freeze–thaw cycle numbers increase, the fracture energies of the specimens exposed to NaCl solutions of different concentrations all decrease to varying degrees. This shows that the damage in the rubber asphalt–aggregate system accumulates gradually during the salt–freeze–thaw process, thus reducing the energy dissipation capacity of the specimens upon failure. Toward the end of processing procedure, the rate of fracture energy attenuation slows, and the values eventually become stable. For example, in a 6% NaCl solution, the fracture energy declines by 20.18%, 28.64%, 37.46%, 48.07%, and 51.57% after 3, 6, 9, 12, and 15 action times, respectively. Evidently, as the number of action times rises, the degradation impact of salt–freeze–thaw on asphalt materials and the accelerating effect on aging gradually weaken. Consistent with the pattern of pull-out strength, the fracture energy between rubber asphalt and the aggregate combination also decreases steadily with higher NaCl concentrations. At lower NaCl concentrations, especially below 6%, the deterioration rate of fracture energy is significantly faster. This occurs because the salt solution concentration plays a crucial role during the initial processing process. When the concentration rises, the quantities of Na^+^ and Cl^−^ ions in the solution increase accordingly. These highly polarizable salt ions actively displace asphalt from aggregate surfaces, resulting in weakened interfacial bonding. Nevertheless, as the quantity of coupled salt–freeze–thaw cycles grows, the damage process progressively stabilizes. The observed stabilization at higher action numbers also suggests that the degradation process is self-limiting. Once the accessible interfacial zones are sufficiently compromised and the asphalt film becomes pervasively aged, the driving force for further rapid deterioration diminishes, leading to the observed plateau in fracture energy loss [61].

### 3.2. Interfacial Salt Damage Mechanism Between Rubber Asphalt and Aggregate

In salt-corroded conditions, the microscopic characteristics of rubber asphalt are critical factors that impact its macroscopic bonding performance with aggregates. To thoroughly decipher the damage mechanism of salt erosion for the rubber asphalt–aggregate system, AFM and FTIR tests were carried out. These tests were designed to examine the microscopic morphological features and the variation laws of chemical functional groups in rubber asphalt. Meanwhile, a model of the rubber asphalt–solution–aggregate was developed via MD simulations to explore the interfacial behavior between rubber asphalt and aggregates during the salt erosion process at the molecular scale.

#### 3.2.1. Microscopic Properties of Rubber Asphalt

##### Microscopic Morphology of Rubber Asphalt After Salt–Freeze–Thaw Cycles

After different freeze–thaw cycles while exposed to 6% NaCl solution, the microscopic morphology of rubber asphalt is illustrated in Table 3. The matrix asphalt was selected as a control group as well.

Table 3 reveals distinct microstructural characteristics between the matrix asphalt and rubber asphalt. Matrix asphalt features a multitude of uniformly distributed “bee-like structures” (usually with a diameter ranging from 1 to 3 μm), and the individual areas of these structures are relatively small. In sharp contrast, the rubber asphalt displays an almost indiscernible honeycomb morphology, which suggests that the incorporation of rubber powder significantly modifies the asphalt’s microstructure. When rubber powder is blended into the asphalt, a three-dimensional network is formed. This structure adsorbs asphaltene and restricts its interaction with waxy components, thereby inhibiting the formation of the honeycomb structure. After the rubber asphalt undergoes the salt–freeze–thaw process, the surface morphology undergoes pronounced changes. The evolution of the honeycomb structure may be divided into three distinct phases: (1) Initial Stage: Numerous irregular pits and grooves emerge on the surface of the rubber asphalt, along with the formation of nascent bee-like structures. (2) Intermediate Stage: The quantity of bee-like structures initially rises and then declines, while their average size gradually enlarges. (3) Final Stage: The bee-like structures break down, resulting in a rebound in their quantity. Notably, after 15 freeze–thaw cycles, large bee-like structures fragment into smaller and more uniform units. The height of these structures is reduced, and the phase contrast between the “bee-like” and “non-bee” regions weakens. This phenomenon reflects the progressive homogenization of the asphalt’s multiphase components, accompanied by a decrease in the surface microstructural undulation. Consequently, the morphology of rubber asphalt increasingly resembles that of the matrix asphalt. After the freeze–thaw process, the rapid temperature drops cause a substantial slowdown in molecular diffusion. As a result, the molecules are unable to relax into the lowest energy configurations and instead undergo local phase transitions, which are manifested as the fragmentation of large bee-like structures into smaller ones. This explains why repeated salt–freeze–thaw processes gradually diminish the variation in the microstructural features between the rubber asphalt and matrix asphalt.

**Figure 11:**
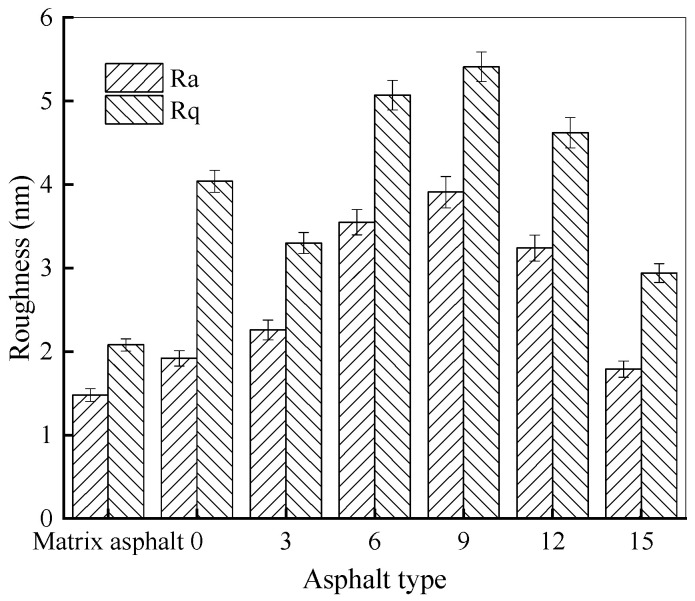
The roughness of rubber asphalt after freeze–thaw cycles with different salts.

Furthermore, Figure 11 presents the roughness parameters of the rubber asphalt after being subjected to various freeze–thaw cycles in a 6% NaCl solution.

Figure 11 demonstrates that untreated rubber asphalt has higher surface roughness than matrix asphalt. Subjected to the freeze–thaw process in a 6% NaCl solution, rubber asphalt experiences significant changes in surface roughness. Both the Ra and Rq parameters follow a distinct non-monotonic trend, initially increasing and then decreasing, with maximum roughness values after nine salt–freeze–thaw cycles. At this stage, the Ra and Rq values increase by 103.65% and 63.94%, respectively, compared with the untreated rubber asphalt of the control group. This suggests that the microstructure of rubber asphalt reorganizes during the intermediate salt–freeze–thaw process. However, after 15 action times, both roughness parameters drop below the levels of the control group, remaining only slightly higher than those of matrix asphalt. These changes in roughness are well correlated with the observed alterations in the morphology of bee-like structures through AFM analysis, reflecting the quantitative evolution of these features in the salt–freeze–thaw process. The effect of salt and freeze–thaw accelerates the migration of components in rubber asphalt, causing its surface characteristics to gradually resemble those of matrix asphalt.

The results suggest that short-term freeze–thaw exposure can enrich the surface morphology of rubber asphalt. During initial cycles, light components are converted into asphaltene. This is consistent with Masad et al.’s observation that asphaltene content influences the generation of bee-like structures [62]. Furthermore, the thermal stress from freeze–thaw cycles accelerates the diffusion and reorganization of waxes and other crystalline components within the asphalt, further contributing to the initial increase in roughness [63]. As the number of action times exceeds nine, NaCl crystallization occurs within the asphalt binder. This phenomenon promotes the progressive leaching of weakly polar components (particularly saturates and aromatics), leading to microstructural degradation. The gradual breakdown of bee-like structures results in two significant morphological changes: a substantial decrease in surface roughness and gradual convergence toward the morphological characteristics of matrix asphalt. These microstructural transformations ultimately result in reduced bonding performance of the rubber asphalt and aggregates, highlighting the critical relationship between surface morphology and interfacial bonding properties.

##### Chemical Functional Groups of Rubber Asphalt After Salt–Freeze–Thaw Cycles

**Figure 12:**
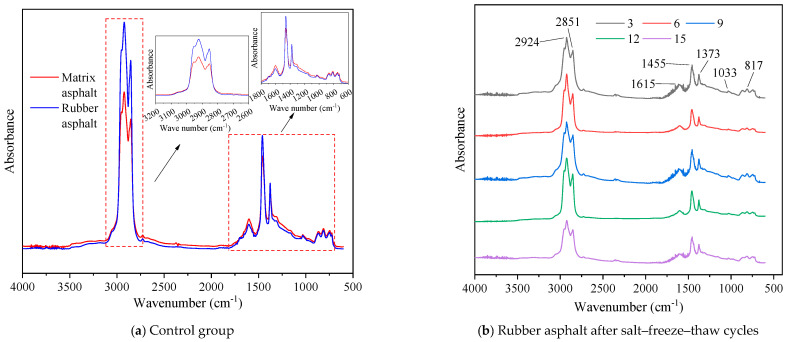
Infrared spectrum of rubber asphalt under the coupling effect of salt–freeze–thaw cycles.

The infrared spectrum of rubber asphalt treated with various freeze–thaw cycles in 6% NaCl solution is illustrated in Figure 12.

As depicted in Figure 12, the main absorption peak locations are almost identical in the infrared spectrum of rubber asphalt and matrix asphalt from the control group (not exposed to salt–freeze–thaw cycles), with only slight variations in the intensity of certain peaks. For rubber asphalt, within the functional group regions, the most prominent peaks are located at approximately 2920 cm^−1^ and 2852 cm^−1^. These peaks relate to the asymmetric and symmetric stretching vibrations of the alkane methylene group, with a wavenumber difference of approximately 68 cm^−1^. Other characteristic peaks include the C=O stretching vibration around 1615 cm^−1^, the –CH_2_– scissoring vibration at 1455 cm^−1^, a broad band around 1373 cm^−1^ assigned to O–H bending vibrations, and the S=O stretching vibration at 1033 cm^−1^.

When exposed to the combined salt–freeze–thaw effect, significant changes are observed in the intensities of typical absorption peaks in the infrared spectrum of rubber asphalt. Notably, the intensities of the peaks around 2924 cm^−1^ and 2851 cm^−1^, corresponding to the stretching vibrations of alkane methylene, decrease substantially. This implies that the salt–freeze–thaw effect lowers the content of saturated components. More pronounced alterations are seen in both the shapes and areas of the absorption peaks at 1615 cm^−1^ and 1033 cm^−1^. These changes indicate oxidative transformations, where unsaturated chains and cycloalkanes in the rubber asphalt are converted into aldehyde and ketone functional groups, leading to expanded areas of carbonyl peaks. Meanwhile, the oxidation of sulfur-containing compounds results in the formation of sulfoxides, as evidenced by the enhanced intensity of the sulfoxide peak. These findings align with the existing literature, which demonstrates that the salt–freeze–thaw cycles promote asphalt aging [47]. Consequently, the typical peaks around 1615 cm^−1^ and 1033 cm^−1^ are confirmed as effective markers for evaluating salt–freeze–thaw-induced damage to rubber asphalt.

**Figure 13:**
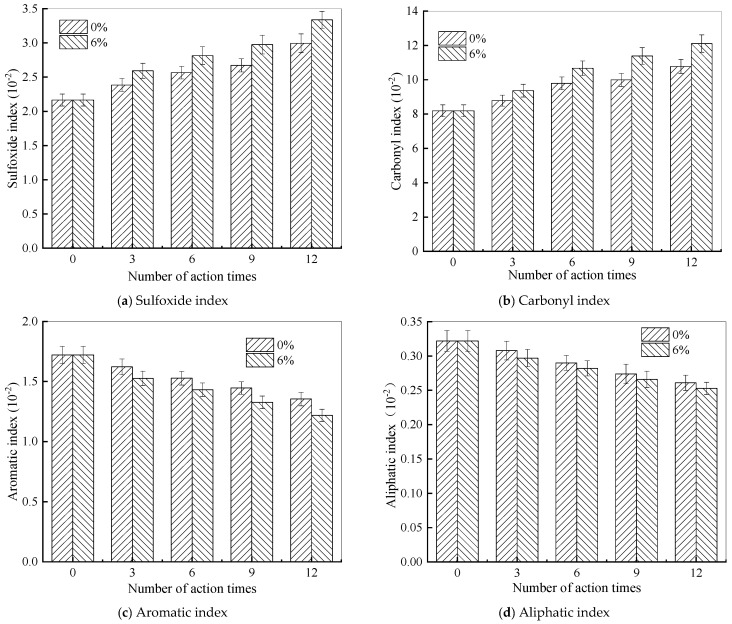
Functional group indexes of rubber asphalt under salt–freeze–thaw coupling effect.

Furthermore, the indexes of chemical functional groups for rubber asphalt following various freeze–thaw cycles in a 6% NaCl solution are presented in Figure 13.

As illustrated in Figure 13, following the freeze–thaw process in water and a 6% NaCl solution, the sulfoxide and carbonyl indexes of rubber asphalt gradually increase as the quantity of freeze–thaw cycles grows. Conversely, the aromatic index and aliphatic index show a downward trend with more action times. Moreover, for the same freeze–thaw cycles, the variation ranges of each index in the 6% NaCl solution are more significant compared to pure water. This further validates the detrimental effect of salt solutions on the properties of rubber asphalt. Taking 15 action times as an example, compared with the control group in water and the 6% NaCl solution, the sulfoxide index of rubber asphalt rises by 44.09% and 64.92%, respectively, the carbonyl index increases by 37.12% and 54.24%, respectively, the aromatic index decreases by 25.26% and 35.97%, respectively, and the aliphatic index decreases by 21.55% and 25.78%, respectively. Under the same test conditions, the amplitudes of variation in the sulfoxide index and the carbonyl index are larger than those in the aromatic index and the aliphatic index.

In a salt erosion environment, the sulfoxide index of rubber asphalt steadily rises as the number of freeze–thaw cycles increases. Hydroperoxides in the salt solution act as oxidants, reacting with sulfur-containing groups in rubber asphalt and increasing the sulfoxide content. Meanwhile, this environment also accelerates the aging process of rubber asphalt, generating substances with C-O bonds and carbonyl groups, which are further oxidized to carboxylic acids during salt freezing. Due to the salt–freeze–thaw process, salt-induced aging in rubber asphalt reduces the solubility of non-polar or weakly polar molecules, resulting in fewer aromatic components, more asphaltene and resin, and a decline in the aromatic index. As the quantity of salt–freeze–thaw cycles rises, the aliphatic index of rubber asphalt decreases. Similar to the variation in the aromatic index, the “salt aging” effect of chlorides intensifies, resulting in a reduction in the saturate content in rubber asphalt.

Variations in these functional group indexes are the primary factors contributing to the microstructural changes in rubber asphalt after the combined action of salt and freeze–thaw cycles. These changes not only influence the physical characteristics of rubber asphalt, such as surface roughness, but may also have a profound impact on its performance and long-term durability during application. To summarize, the degree to which rubber asphalt deteriorates after the salt–freeze–thaw process can be assessed by monitoring changes in the carbonyl and sulfoxide indexes.

#### 3.2.2. Molecular Behavior at the Asphalt–Aggregate Interface

##### Diffusion Behavior of H_2_O

**Figure 14:**
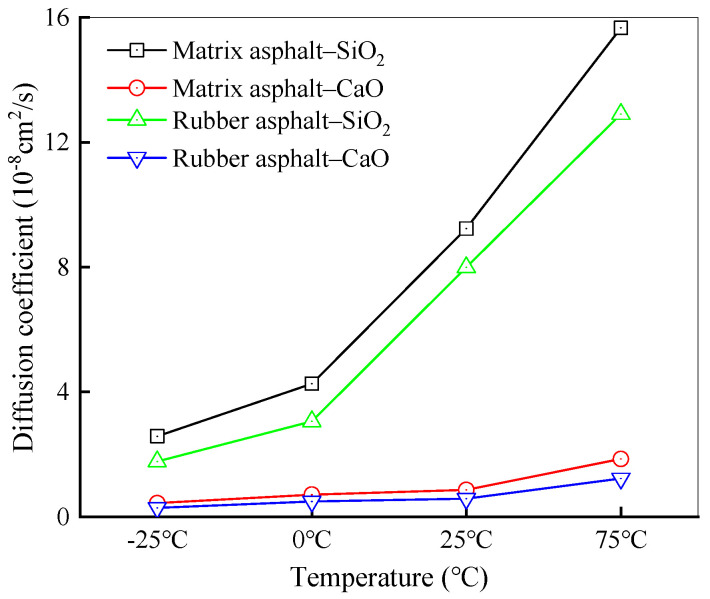
Diffusion coefficients of water molecules across different temperatures.

To explore the salt erosion mechanism between asphalt and aggregates, MD simulations were employed to analyze water molecule diffusion coefficients within the system across various temperatures. The temperatures for simulation were selected as −25 °C, 0 °C, 25 °C, and 75 °C, and the quantity of water molecules in the models was fixed at 100. The diffusion coefficients for water molecules at these various temperatures are presented in Figure 14.

As illustrated in Figure 14, the diffusion coefficients of water molecules in all four models exhibit a distinct upward trend with increasing temperature. As the temperature rises, the molecular thermal motion becomes more vigorous. Moreover, at higher temperatures, the differences in diffusion coefficients for the same components become more prominent. There are multiple reasons contributing to the sharp increase in the diffusion coefficient when the temperature reaches 75 °C. Firstly, higher temperatures enhance the activity of water molecules, causing their volume to expand, which in turn promotes water molecule diffusion. Secondly, higher temperatures weaken the intermolecular interaction forces within the asphalt, reducing its viscosity and thus facilitating the diffusion of water. At 75 °C, among the four systems, the water molecule diffusion coefficient in the matrix asphalt-SiO_2_ system is the largest, reaching 15.66. Conversely, the diffusion coefficient in the rubber asphalt–CaO system is the smallest, standing at 1.229.

In summary, temperature, along with asphalt and aggregate types, is the primary factor influencing water molecule diffusion at the asphalt–aggregate interface. At elevated temperatures, molecular diffusion occurs more readily, and the oxygen atoms in water easily form chemical bonds with the metal ions in the aggregates, which is consistent with the conclusion obtained by Tang et al. and Luo et al. [64,65]. Simultaneously, the metal ions transfer electrons from the aggregate to the water molecules, leading the latter to adhere to the surface of aggregates. Although the interfacial bonding between asphalt and aggregate in salt-eroded environments involves numerous factors, the theory of water molecule diffusion between the two materials enhances the understanding of the interfacial damage mechanism.

**Figure 15:**
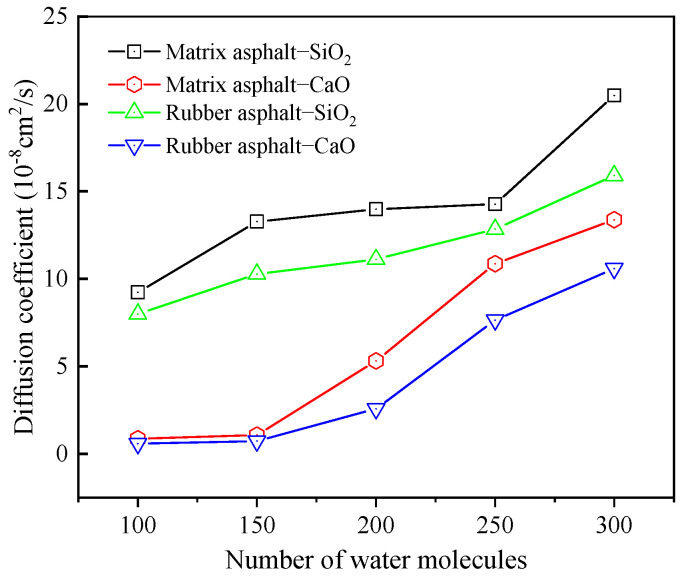
Diffusion coefficients of water molecules under different water molecule numbers.

For a deeper analysis of the impact of water molecule numbers on diffusion properties, the interfacial diffusion coefficients of water molecules within the asphalt–aggregate system were computed across varying quantities of water molecules. The simulation temperature was set at 25 °C, with the findings presented in Figure 15.

As presented in Figure 15, within the matrix asphalt–SiO_2_–water system, the diffusion coefficient experiences an increase of about 51.46% when the quantity of water molecules rises from 100 to 200. Subsequently, when the number further increases from 200 to 300, the diffusion coefficient goes up by approximately 46.57%. This suggests that as the quantity of water molecules grows, the diffusion coefficient also increases accordingly. This discovery is vital for comprehending the behavior of water molecules in complex environments, particularly in systems involving adsorbent materials like SiO_2_. In the case of the matrix asphalt–CaO model, the greater hydrophilicity of CaO causes increased adsorption of water molecules on the aggregates. As a result, the diffusion coefficient of water molecules is smaller when compared with the SiO_2_ model. For the rubber asphalt–SiO_2_ model with water layers, the diffusion coefficient for water molecules rises at a relatively slow rate when the number of water molecules increases. In contrast, the trend observed in the rubber asphalt–CaO mirrors that of matrix asphalt, exhibiting a significant upward trend. The root cause of this phenomenon primarily stems from the specific chemical bonding formed between the oxygen atoms and metal ions. These bonds not only promote the adsorption of water molecules on the interface of CaO but also become stronger as electrons are transferred from the metal ions in CaO to the water molecules. Since the electron content of the metal ions in CaO is fixed, once a portion of electrons is transferred to water molecules, the capacity of these metal ions to transfer electrons to the polar groups in asphalt will be diminished. As a result, water molecules will engage in competition with asphalt for the adsorption sites on the interface with aggregates.

##### Diffusion Behavior of Na and Cl Ions

**Figure 16:**
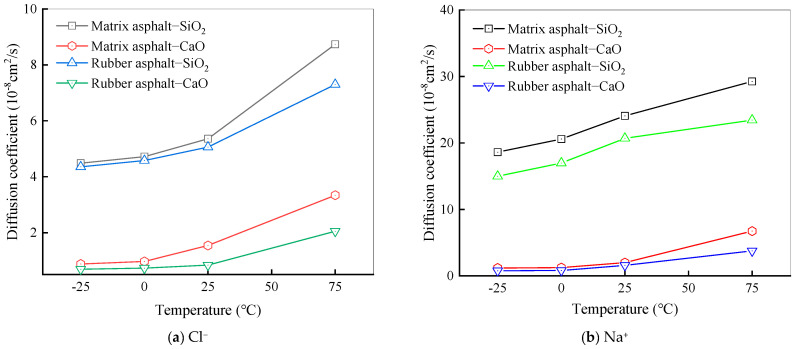
Diffusion coefficients of NaCl ions at different temperatures.

The diffusion coefficients of NaCl ions across various temperatures on matrix asphalt and rubber asphalt surfaces are depicted in Figure 16. At this stage, the quantity of water molecules was kept constant at 100, and the concentration of the salt solution was set at 6%.

As presented in Figure 16, within the low-temperature range (−25 °C to 0 °C), the diffusion coefficients of Na^+^ and Cl^−^ do not display an obvious trend of change with increasing temperature. This implies that the diffusion behavior of Na^+^ and Cl^−^ is relatively stable within this temperature interval. Notably, the change trend for the SiO_2_ model is slightly more pronounced than that for the CaO model, which might be associated with the variations in physical and chemical characteristics between SiO_2_ and CaO. Moreover, as the temperature rises, the diffusion coefficients of sodium ions and chloride ions display a distinct increasing tendency. Particularly in the high-temperature range (25 °C to 75 °C), the diffusion coefficients of Na^+^ and Cl^−^ grow rapidly. As the temperature goes up, the kinetic energy of sodium ions and chloride ions gradually increases, accelerating their movement speed and directly enhancing the diffusion efficiency of particles in the system. In contrast, the particles show significantly inert behavior in environments below 0 °C, which could be ascribed to the restricted movement of particles in low-temperature settings.

From the above analysis, the diffusion behavior of Na^+^ and Cl^−^ exhibits high sensitivity to temperature changes, especially in the high-temperature range (25 °C~75 °C), where the diffusion coefficient increases rapidly. Within the low-temperature interval, the diffusion coefficient values remain relatively stable with a slow increasing trend. According to the stochastic nature of molecular motion, this temperature-dependent diffusion behavior is such that with a rise in temperature, the thermal motion between molecules becomes more vigorous, leading to an increase in the frequency and energy of intermolecular collisions. The enhanced thermal motion enables sodium ions and chloride ions to overcome the constraints of surrounding molecules easily, thereby facilitating diffusion in the solution. Additionally, the increase in temperature also enhances the intermolecular interaction forces. Specifically, elevated temperature weakens the cohesive strength of the asphalt binder, potentially creating more pathways for ion ingress [66]. In the mixing system of asphalt, aggregate, and solution, all types of molecules are affected by the interaction forces of neighboring molecules. This force promotes the aggregation and adsorption of molecular clusters, gradually approaching a stable state. The research of Wang et al. also confirmed this conclusion [67]. Similarly, the diffusion behavior of salt molecules is also affected, thus exhibiting temperature-sensitive diffusion behavior.

**Figure 17:**
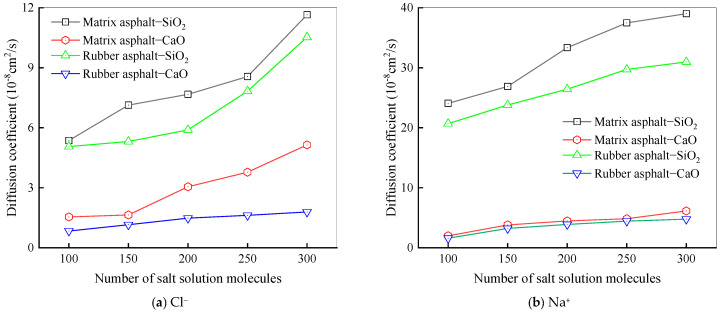
Diffusion coefficients of NaCl ions under different salt solution molecules.

To study how the number of salt solution molecules affects the diffusion behavior of Na^+^ and Cl^−^, the simulation temperature was selected as 25 °C, and the concentration of the salt solution was fixed at 6%. The numbers of molecules in the salt solution were set to 100, 150, 200, 250, and 300, respectively. Figure 17 depicts the diffusion coefficients of salt ions at the asphalt–aggregate interface across varying numbers of salt solution molecules.

As shown in Figure 17, the diffusion coefficients of Na^+^ and Cl^−^ gradually rise with an upward trend in the number of molecules present in the salt solution. Under the same conditions, the diffusion coefficient of Na^+^ exceeds that of Cl^−^, meaning Na^+^ exhibits far more energetic diffusion motion than Cl^−^. This is because metal ions have a greater tendency to accumulate on aggregate surfaces, while chloride ions possess nucleophilic properties, and the polarization-induced effect brings them closer to positively charged atoms within asphalt [68]. For the asphalt–CaO model, Na^+^ accumulates more noticeably on CaO, whereas Cl^−^ is more attracted to asphalt. Negatively charged oxygen atoms on CaO can attract Na^+^, enabling Na^+^ to more readily take up active sites on the CaO surface and incidentally facilitating Cl^−^ diffusion into asphalt.

##### Diffusion Behavior of Four Components in Asphalt

For the rubber asphalt–salt solution molecules–SiO_2_ system, the diffusion coefficients of four components within the rubber asphalt were obtained, as presented in Table 4. The simulation temperature was kept at 25 °C, and the concentration of salt solution was fixed at 6%.

As indicated in Table 4, in the rubber asphalt–100 salt solution molecules–SiO_2_ model, saturates exhibit the highest degree of diffusion, followed by aromatics, asphaltene, and resin. Among the light components, saturates diffuse more readily than aromatics. In the heavy fraction, the diffusion coefficient of asphaltene increases more significantly than that of resin. Obviously, the presence of salt solution molecules between rubber asphalt and the aggregate may facilitate the migration and diffusion of asphaltene and increase its diffusion rate. When the number of salt solution molecules is 200, there is a notable increase in the diffusion coefficients of the four components. Saturates have the highest diffusion coefficient, followed by aromatics and resin, while asphaltene has the lowest. Compared with the 200 salt solution molecule model, the resin diffusion coefficient is higher in the rubber asphalt–300 salt solution molecules–SiO_2_ model. This indicates that while asphaltene reacts quickly to the salt solution, the salt solution exerts a more pronounced driving effect on resin. Additionally, a higher number of salt solution molecules brings about a rise in the diffusion coefficient of asphaltene, which means that the spreading and pushing impact of salt solution molecules on asphaltene becomes prominent when the salt solution content is high. This could be attributed to the increased osmotic stress and competitive polar interactions introduced by the high ion concentration. These factors disrupt the associative forces holding the asphaltene together, thereby enhancing their mobility [69]. Obviously, under salt erosion conditions, the diffusion coefficient of non-polar saturates is markedly enhanced. This phenomenon reveals that Cl^−^ and Na^+^ promote the diffusion of non-polar saturates through their polarization-inducing effects.

#### 3.2.3. Damage Mechanism of Asphalt–Aggregate Under Salt Erosion Conditions

**Figure 18:**
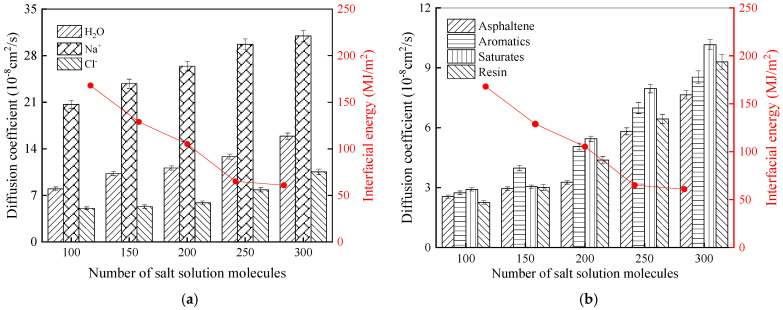
Molecular diffusion and interfacial energy of rubber asphalt–aggregate. (**a**) Molecular diffusion of solution layers and interfacial energy. (**b**) Molecular diffusion of asphalt components and interfacial energy.

To further explore the mechanism of the salt erosion effect, considering the rubber asphalt–SiO_2_ system as an illustration, Figure 18 illustrates the relationships between the variations in interfacial energy and the diffusion coefficients for water molecules, salt ions, and asphalt components under simulated conditions of a temperature of 25 °C and a salt solution concentration of 6%.

As shown in Figure 18, when the molecular number of the salt solution is small, the diffusion coefficients of several constituents are low. In the current stage, due to the limited quantity of salt solution molecules, the invasion degree is relatively low, and the aggregate has not yet reached saturation. As a result, most of the invading solution is adsorbed on the aggregate surface and does not occupy sufficient adsorption sites of rubber asphalt. The interface of rubber asphalt and aggregates remains structurally intact, and the diffusion degrees of both the solution and asphalt are at low levels. However, with the deepening of salt solution intrusion, the diffusion coefficient for salt solution molecules increases abruptly. Correspondingly, there is a reduction in the interfacial energy between rubber asphalt and the aggregate, which indicates that salt solution molecules begin to occupy the adsorption sites on the asphalt surface together with the aggregate. At this point, the weak bond linking rubber asphalt and the aggregate begins to be disrupted by salt solution molecules, resulting in weaker bonding and lower interfacial energy.

**Figure 19:**
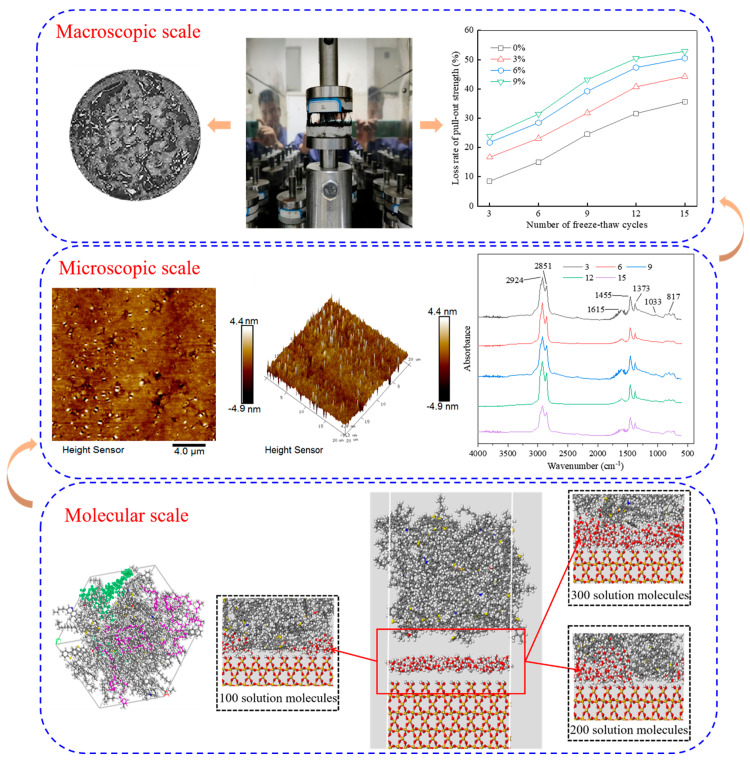
Conceptual model of the multiscale deterioration mechanism of the rubber asphalt–aggregate interface under salt erosion conditions.

To synthesize the findings from macro-mechanical tests, micro-characterization, and MD simulations, an integrated multiscale deterioration mechanism is proposed, as illustrated in Figure 19. The salt-induced damage is initiated at the molecular scale and propagates to the macroscale, leading to the eventual failure of the interfacial bonding.

As depicted in Figure 19, at the molecular scale, the ingress of water and salt ions (Na^+^ and Cl^−^) into the asphalt–aggregate interface initiates a process of competitive adsorption. The polar water molecules and Na^+^ ions preferentially adsorb onto the active sites of the aggregate surface, displacing the asphalt molecules. This displacement is facilitated by the stronger acid–base interactions between the aggregate surface and the salt solution compared to those with the asphalt. Concurrently, the ions, particularly Na^+^, which exhibits higher mobility than Cl^−^, disrupt the internal structure of the asphalt, which weakens the cohesive interactions within the asphalt binder and, through polarization effects, preferentially enhances the mobility of non-polar saturates [70]. This leads to an overall increase in the diffusion coefficients of all asphalt components, as shown in Table 4, signifying a plasticization and destabilization of the asphalt film at the interface.

These molecular-scale events manifest as observable changes at the microscopic scale. FTIR analysis confirms the occurrence of “salt aging”, characterized by a significant increase in carbonyl and sulfoxide indexes and a decrease in aliphatic and aromatic indexes. This chemical hardening and oxidation reduce the asphalt’s flexibility and self-healing capability. AFM results provide direct evidence of the corresponding microstructural evolution. The initial reorganization and subsequent breakdown of bee-like structures, along with the non-monotonic change in surface roughness, reflect the progressive deterioration of the micromechanical properties of rubber asphalt. The morphology of rubber asphalt gradually converges toward that of the more brittle, aged matrix asphalt.

The culmination of these chemical and morphological degradations at the microscopic scale directly leads to the loss of macroscopic pull-out strength. The combined effects of (a) adhesive failure due to competitive displacement at the interface, (b) cohesive weakening of the asphalt film due to salt aging and component migration, and (c) physical damage from salt crystallization pressure during freeze–thaw cycles result in a severe reduction in interfacial bonding properties. This is quantitatively measured as a dramatic decline in pull-out strength and fracture energy. The interface loses its integrity, leading to debonding, which macroscopically manifests as stripping and the formation of micro-cracks in the asphalt mixture [71].

In summary, the multiscale investigation establishes a clear cause–effect chain: molecular competitive adsorption and ion diffusion, microscopic chemical aging and morphological homogenization, and macroscopic degradation of interfacial mechanical properties. This integrative understanding is crucial for developing more durable asphalt materials for salt-eroded environments.

## 4. Conclusions

Higher concentrations of NaCl solution and more freeze–thaw cycles result in decreased pull-out strength and fracture energy between rubber asphalt and various aggregates. Limestone aggregate demonstrates superior bonding performance with rubber asphalt, followed by basalt and granite, verifying that alkaline aggregates adhere better to asphalt than acidic ones in salt erosion environments. After 15 freeze–thaw cycles while exposed to 6% NaCl solution, the pull-out strength and fracture energy of the rubber asphalt–limestone aggregate decrease by 50.47% and 51.57%, respectively. Moreover, after 12 salt–freeze–thaw cycles, the effect of NaCl crystallization on the damage of the asphalt film at the sample interface and the aging process is limited. The decline in pull-out strength of the rubber asphalt–aggregate system decelerates and then reaches a stable state.

Salt–freeze–thaw cycles cause significant microstructural changes in rubber asphalt, creating surface pits and partial regeneration of bee-like structures. Long-term exposure leads to phase homogenization, reducing surface roughness and making the morphology similar to matrix asphalt. The coupled environmental effect changes peak intensities of the FTIR spectrum while keeping the chemical composition. Following 15 freeze–thaw cycles in 6% NaCl solution, rubber asphalt exhibits 65.42% and 52.34% increases in the carbonyl and sulfoxide indexes, respectively, contrasted by 49.24% and 42.5% decreases in the aromatic and aliphatic indexes. This accelerates the aging of rubber asphalt, evidenced by rising carbonyl/sulfoxide indexes and declining aliphatic/aromatic indexes. The sulfoxide and carbonyl indexes vary more significantly than the aromatic and aliphatic indexes under identical conditions.

The diffusion of water at the interface of the rubber asphalt–aggregate is temperature-dependent. Compared to SiO_2_ surfaces, CaO surfaces exhibit stronger water adsorption capability and lower molecular mobility. In salt erosion environments, Na^+^ migrates faster at the interface than Cl^−^, which indicates its dominant role in salt-induced deterioration. The asphalt components have varying diffusion rates, with lighter saturates being more mobile than aromatics, resin, and asphaltene. A higher content of salt solution promotes the diffusion of these components, suggesting that salt and moisture weaken interfacial bonding. Notably, Na^+^ and Cl^−^ ions preferentially facilitate the transport of saturate molecules at the interface.

The salt–freeze–thaw effect severely deteriorates the rubber asphalt–aggregate interfacial bonding through synergistic physicochemical mechanisms. Accelerated aging is manifested by significant increases in carbonyl and sulfoxide indexes and microstructural evolution toward matrix asphalt. At the interface, the faster mobility of Na^+^ than Cl^−^ leads to its preferential accumulation on aggregate surfaces. The competitive adsorption between salt ions/water molecules and asphalt components disrupts the original bonding. This interfacial displacement increases the diffusion coefficients for asphalt components, causing adhesive weakening and asphalt film detachment. The combined influence of accelerated aging, salt crystallization, and enhanced ionic diffusion drives the deterioration of interfacial bonding, explaining the reduction in macroscopic pull-out strength under harsh conditions.

The perspectives of this work involve expanding the research scope to cover more types of modified asphalts and aggregates. Additionally, this involves simulating complex on-site environments, such as the coupling of salt, UV radiation, and vehicle loads. Furthermore, the multiscale correlation models will be optimized to better understand the relationships across different scales. The mix designs for cold regions will also be guided through on-site validation, ensuring that the designs are practical and effective. Finally, evaluation standards for salt erosion resistance will be established to enhance the durability of asphalt pavements in cold regions.

## Figures and Tables

**Table 1:** Fundamental performance parameters of rubber asphalt.

Index	Tested Value		Standard
	Matrix Asphalt	Rubber Asphalt	
Penetration (25 °C,100 g, 5 s, 0.1 mm)	64	42.3	ASTM D5 [42]
Ductility (15 °C, 5 mm/min, cm)	>100	10.47	ASTM D113 [43]
Softening point/°C	48.0	72.6	ASTM D36 [44]
135 °C Brookfield viscosity/Pa▪S	1.872	9.125	ASTM D2196 [45]
RTFO treated at 163C for 85 min			
Penetration (25 °C, 100 g, 5 s, 0.1 mm)	63.9	63.9	ASTM D5 [42]
Ductility (15 °C, 5 mm/min, cm)	8.5	8.5	ASTM D113 [43]

**Table 2:** Main chemical compositions of three kinds of aggregates.

Aggregate Type	Chemical Composition (%)				
	SiO_2_	CaO	Al_2_O_3_	K_2_O	Fe_2_O_3_
Limestone	8.1	65.2	3.6	3.0	2.4
Basalt	46.7	9.3	15.2	2.9	11.5
Granite	66.5	3.2	14.9	4.1	3.8

**Table 3:** AFM morphology of rubber asphalt after salt–freeze–thaw effect.

Asphalt Type	Morphology Characteristic	
	2D	3D
Matrix asphalt	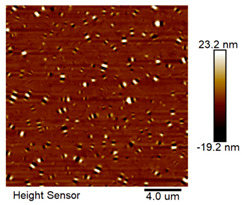	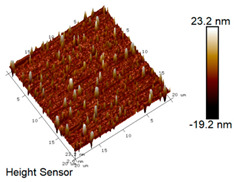
Rubber asphalt	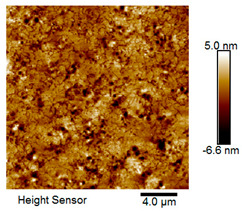	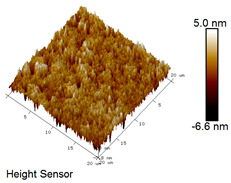
Rubber asphalt after 3 freeze–thaw cycles	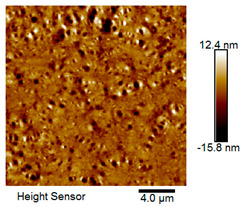	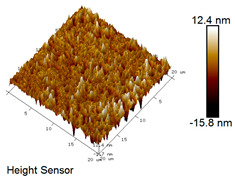
Rubber asphalt after 6 freeze–thaw cycles	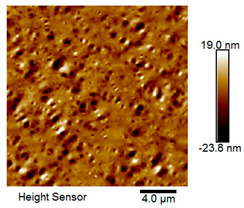	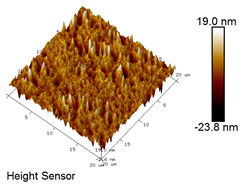
Rubber asphalt after 9 freeze–thaw cycles	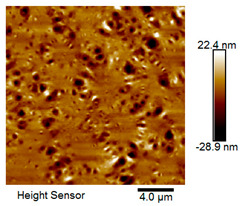	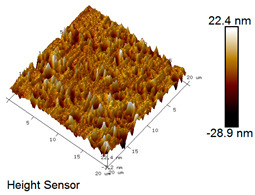
Rubber asphalt after 12 freeze–thaw cycles	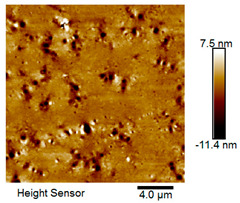	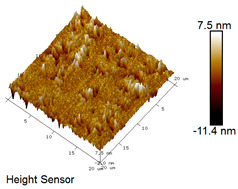
Rubber asphalt after 15 freeze–thaw cycles	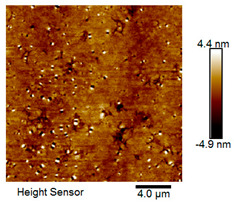	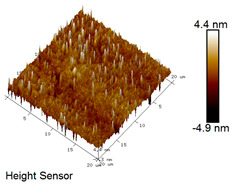

**Table 4:** Diffusion coefficients of four components in the rubber asphalt.

Number of Salt Solution Molecules	Diffusion Coefficient (10^−8^ cm^2^/s)			
	Asphaltene	Aromatics	Saturates	Resin
100	2.55	2.74	2.91	2.26
150	2.95	3.98	3.05	3.01
200	3.27	5.06	5.45	4.38
250	5.82	6.99	7.96	6.44
300	7.65	8.54	10.16	9.3

## Data Availability

The original contributions presented in this study are included in the article. Further inquiries can be directed to the corresponding author.
